# Supplementary material for: Bayesian refinement of protein structures and ensembles against SAXS data using molecular dynamics
Source: PLoS Comput Biol. 2017 Oct 18;13(10):e1005800. doi: 10.1371/journal.pcbi.1005800 (PMC5662244; doi:10.1371/journal.pcbi.1005800)
Supplement: S1 Archive — (BZ2) [file pcbi.1005800.s011.tar.bz2 › gromacs_4.62_bayesian_saxs_refi/share/html/online/mdp_opt.html]

mdp options


| mdp options Main Table of Contents |

**VERSION 4.6  
Sat 19 Jan 2013**

---


### Table of Contents

- **General remarks**- **preprocessing** (include, define)- **run control** (integrator, tinit, dt, nsteps, init-step, comm-mode, nstcomm, comm-grps)- **langevin dynamics** (bd-fric, ld-seed)- **energy minimization** (emtol, emstep, nstcgsteep)- **shell molecular dynamics**(emtol,niter,fcstep)- **test particle insertion**(rtpi)- **output control** (nstxout, nstvout, nstfout, nstlog, nstcalcenergy, nstenergy, nstxtcout, xtc-precision, xtc-grps, energygrps)- **neighbor searching** (cutoff-scheme, nstlist, nstcalclr, ns-type, pbc, periodic-molecules, verlet-buffer-drift, rlist, rlistlong)- **electrostatics** (coulombtype, coulomb-modifier, rcoulomb-switch, rcoulomb, epsilon-r, epsilon-rf)- **VdW** (vdwtype, vdw-modifier, rvdw-switch, rvdw, DispCorr)- **tables** (table-extension, energygrp-table)- **Ewald** (fourierspacing, fourier-nx, fourier-ny, fourier-nz, pme-order, ewald-rtol, ewald-geometry, epsilon-surface, optimize-fft)- **Temperature coupling** (tcoupl, nsttcouple, tc-grps, tau-t, ref-t)- **Pressure coupling** (pcoupl, pcoupltype,
                              nstpcouple, tau-p, compressibility, ref-p, refcoord-scaling)- **simulated annealing** (annealing, annealing-npoints, annealing-time, annealing-temp)- **velocity generation** (gen-vel, gen-temp, gen-seed)- **bonds** (constraints, constraint-algorithm, continuation, shake-tol, lincs-order, lincs-iter, lincs-warnangle, morse)- **Energy group exclusions** (energygrp-excl)- **Walls** (nwall, wall-type, wall-r-linpot, wall-atomtype,
                                        wall-density, wall-ewald-zfac)- **COM pulling** (pull, ...)- **NMR refinement** (disre, disre-weighting, disre-mixed, disre-fc, disre-tau, nstdisreout, orire, orire-fc, orire-tau, orire-fitgrp, nstorireout)- **Free energy calculations** (free-energy, nstdhdl, dhdl-print-energy, init-lambda, delta-lambda, fep-lambdas, coul-lambdas, vdw-lambdas, bonded-lambdas, restraint-lambdas, mass-lambdas, temperature-lambdas, sc-alpha, sc-coul, sc-power, sc-r-power, sc-sigma, couple-moltype, couple-lambda0, couple-lambda1, couple-intramol)- **Expanded ensemble simulation** (lmc-stats, lmc-mc-move, lmc-seed, lmc-gibbsdelta, mc-temperature, nst-transition-matrix, init-lambda-weights, initial-wl-delta, wl-scale, wl-ratio, symmetrized-transition-matrix, lmc-forced-nstart, mininum-var-min, lmc-weights-equil, weight-equil-wl-delta, weight-equil-number-all-lambda, weight-equil-number-steps, weight-equil-number-samples, weight-equil-count-ratio, simulated-tempering, simulated-tempering-scaling, sim-temp-low, sim-temp-high)- **Non-equilibrium MD** (acc-grps, accelerate, freezegrps, freezedim, cos-acceleration, deform)- **Electric fields** (E-x, E-xt, E-y, E-yt, E-z, E-zt )- **Mixed quantum/classical dynamics** (QMMM, QMMM-grps, QMMMscheme, QMmethod, QMbasis, QMcharge, Qmmult, CASorbitals, CASelectrons, SH)- **Implicit solvent** (implicit-solvent, gb-algorithm, nstgbradii, rgbradii, gb-epsilon-solvent, gb-saltconc, gb-obc-alpha, gb-obc-beta, gb-obc-gamma, gb-dielectric-offset, sa-algorithm, sa-surface-tension)- **AdResS settings** (adress, adress\_type, adress\_const\_wf, adress\_ex\_width, adress\_hy\_width, adress\_ex\_forcecap, adress\_interface\_correction, adress\_site, adress\_reference\_coords, adress\_tf\_grp\_names, adress\_cg\_grp\_names)- **User defined thingies** (user1-grps, user2-grps, userint1, userint2, userint3, userint4, userreal1, userreal2, userreal3, userreal4)- **Index**


---

  

### General

Default values are given in parentheses. The first option in
the list is always the default option. Units are given in
square brackets The difference between a dash and an underscore
is ignored.

A sample .mdp file is
available. This should be appropriate to start a normal
simulation. Edit it to suit your specific needs and desires.

  


---

### Preprocessing

**include:**
:   directories to include in your topology. Format:

    ```
    -I/home/john/mylib -I../otherlib
    ```

**define:**
:   defines to pass to the preprocessor, default is no defines. You can use
    any defines to control options in your customized topology files. Options
    that are already available by default are::   -DFLEXIBLE
        :   Will tell grompp to include flexible water in stead of rigid water into your
            topology, this can be useful for normal mode analysis.

        -DPOSRES
        :   Will tell grompp to include posre.itp into your topology, used for
            position restraints.

  


---

### Run control

**integrator:** (Despite the name, this list includes algorithms that are not actually integrators. steep and all entries following it are in this category)
:   **md**
    :   A leap-frog algorithmleap-frog integrator
        for integrating Newton's equations of motion.

    **md-vv**
    :   A velocity Verlet algorithm for integrating Newton's equations of motion.
        For constant NVE simulations started from corresponding points in the same trajectory, the trajectories
        are analytically, but not binary, identical to the **md** leap-frog integrator. The the kinetic
        energy, which is determined from the whole step velocities and is therefore
        slightly too high. The advantage of this integrator is more accurate,
        reversible Nose-Hoover and Parrinello-Rahman coupling integration
        based on Trotter expansion, as well as (slightly too small) full step velocity
        output. This all comes at the cost off extra computation, especially with
        constraints and extra communication in parallel. Note that for nearly all
        production simulations the **md** integrator is accurate enough.

    **md-vv-avek**
    :   A velocity Verlet algorithm identical to **md-vv**, except that
        the kinetic energy is determined as the average of
        the two half step kinetic energies as in the **md** integrator, and this thus more accurate.
        With Nose-Hoover and/or Parrinello-Rahman coupling this comes with
        a slight increase in computational cost.

    **sd**
    :   An accurate leap-frog stochastic dynamics integrator.
        Four Gaussian random number are required
        per integration step per degree of freedom. With constraints,
        coordinates needs to be constrained twice per integration step.
        Depending on the computational cost of the force calculation,
        this can take a significant part of the simulation time.
        The temperature for one or more groups of atoms
        (**tc-grps**)
        is set with **ref-t** [K],
        the inverse friction constant for each group is set with
        **tau-t** [ps].
        The parameter **tcoupl** is ignored.
        The random generator is initialized with **ld-seed**.
        When used as a thermostat, an appropriate value for **tau-t** is 2 ps,
        since this results in a friction that is lower than the internal friction
        of water, while it is high enough to remove excess heat
        (unless **cut-off** or **reaction-field** electrostatics is used).
        NOTE: temperature deviations decay twice as fast as with
        a Berendsen thermostat with the same **tau-t**.

    **sd1**
    :   An efficient leap-frog stochastic dynamics integrator.
        This integrator is equivalent to **sd**, except that it requires
        only one Gaussian random number and one constraint step and is therefore
        significantly faster. Without constraints the accuracy is the same as **sd**.
        With constraints the accuracy is significantly reduced, so then **sd**
        will often be preferred.

    **bd**
    :   An Euler integrator for Brownian or position Langevin dynamics, the
        velocity is the force divided by a friction coefficient
        (**bd-fric** [amu ps^-1^])
        plus random thermal noise (**ref-t**).
        When **bd-fric**=0, the friction coefficient for each
        particle is calculated as mass/**tau-t**, as for the
        integrator sd.
        The random generator is initialized with **ld-seed**.

    **steep**
    :   A steepest descent algorithm for energy
        minimization. The maximum step size is **emstep**
        [nm], the tolerance is **emtol** [kJ
        mol^-1^ nm^-1^].

    **cg**
    :   A conjugate gradient algorithm for energy
        minimization, the tolerance is **emtol** [kJ mol^-1^
        nm^-1^]. CG is more efficient when a steepest descent step
        is done every once in a while, this is determined by
        **nstcgsteep**.
        For a minimization prior to a normal mode analysis, which requires
        a very high accuracy, GROMACS should be compiled in double precision.

    **l-bfgs**
    :   A quasi-Newtonian algorithm for energy minimization
        according to the low-memory Broyden-Fletcher-Goldfarb-Shanno approach.
        In practice this seems to converge faster than Conjugate Gradients, but due
        to the correction steps necessary it is not (yet) parallelized.

    **nm**
    :   Normal mode analysisnormal-mode analysis is performed
        on the structure in the tpr file. GROMACS should be
        compiled in double precision.

    **tpi**
    :   Test particle insertion. The last molecule in the topology
        is the test particle. A trajectory should be provided with
        the -rerun option of mdrun. This trajectory
        should not contain the molecule to be inserted. Insertions
        are performed **nsteps** times in each frame at random locations
        and with random orientiations of the molecule. When **nstlist**
        is larger than one, **nstlist** insertions are performed
        in a sphere with radius **rtpi**
        around a the same random location using the same neighborlist
        (and the same long-range energy when **rvdw** or **rcoulomb**>**rlist**,
        which is only allowed for single-atom molecules).
        Since neighborlist construction is expensive, one can perform several
        extra insertions with the same list almost for free.
        The random seed is set with **ld-seed**.
        The temperature for the Boltzmann weighting is set with
        **ref-t**, this should match the temperature
        of the simulation of the original trajectory.
        Dispersion correction is implemented correctly for tpi.
        All relevant quantities are written to the file specified with
        the -tpi option of mdrun.
        The distribution of insertion energies is written to the file specified with
        the -tpid option of mdrun.
        No trajectory or energy file is written.
        Parallel tpi gives identical results to single node tpi.
        For charged molecules, using PME with a fine grid is most accurate
        and also efficient, since the potential in the system only needs
        to be calculated once per frame.

    **tpic**
    :   Test particle insertion into a predefined cavity location.
        The procedure is the same as for **tpi**, except that one coordinate
        extra is read from the trajectory, which is used as the insertion location.
        The molecule to be inserted should be centered at 0,0,0. Gromacs does
        not do this for you, since for different situations a different
        way of centering might be optimal.
        Also **rtpi** sets the radius for the sphere
        around this location. Neighbor searching is done only once per frame,
        **nstlist** is not used.
        Parallel tpic gives identical results to single node tpic.

    **tinit: (0) [ps]**
    :   starting time for your run (only makes sense for integrators md,
        sd and bd)

    **dt: (0.001) [ps]**
:   time step for integration (only makes sense for integrators md,
    sd and bd)

**nsteps: (0)**
:   maximum number of steps to integrate or minimize, -1 is no maximum

**init-step: (0)**
:   The starting step.
    The time at an step i in a run is calculated as: t = tinit + dt\*(init-step + i).
    The free-energy lambda is calculated as: lambda = init-lambda + delta-lambda\*(init-step + i).
    Also non-equilibrium MD parameters can depend on the step number.
    Thus for exact restarts or redoing part of a run it might be necessary to
    set init-step to the step number of the restart frame.
    tpbconv does this automatically.

**comm-mode:**
:   **Linear**
    :   Remove center of mass translation

    **Angular**
    :   Remove center of mass translation and rotation around the center of mass

    **None**
    :   No restriction on the center of mass motion

**nstcomm: (100) [steps]**
:   frequency for center of mass motion removal

**comm-grps:**
:   group(s) for center of mass motion removal, default is the whole system

  


---

### Langevin dynamics

**bd-fric: (0) [amu ps^-1^]**
:   Brownian dynamics friction coefficient.
    When **bd-fric**=0, the friction coefficient for each
    particle is calculated as mass/**tau-t**.

**ld-seed: (1993) [integer]**
:   used to initialize random generator for thermal noise
    for stochastic and Brownian dynamics.
    When **ld-seed** is set to -1, the seed is calculated from the process ID.
    When running BD or SD on multiple processors, each processor uses a seed equal
    to **ld-seed** plus the processor number.

  


---

### Energy minimizationenergy minimization

**emtol: (10.0) [kJ mol^-1^ nm^-1^]**
:   the minimization is converged when the maximum force is smaller than
    this value

**emstep: (0.01) [nm]**
:   initial step-size

**nstcgsteep: (1000) [steps]**
:   frequency of performing 1 steepest descent step while doing
    conjugate gradient energy minimization.

**nbfgscorr: (10)**
:   Number of correction steps to use for L-BFGS minimization. A higher
    number is (at least theoretically) more accurate, but slower.

  


---

### Shell Molecular Dynamicsshell molecular dynamics

When shells or
flexible constraints are present in the system the positions of the shells
and the lengths of the flexible constraints are optimized at
every time step until either the RMS force on the shells and constraints
is less than emtol, or a maximum number of iterations (niter) has been reached

**emtol: (10.0) [kJ mol^-1^ nm^-1^]**
:   the minimization is converged when the maximum force is smaller than
    this value. For shell MD this value should be 1.0 at most, but since the
    variable is used for energy minimization as well the default is 10.0.

**niter: (20)**
:   maximum number of iterations for optimizing the shell positions
    and the flexible constraints.

**fcstep: (0) [ps^2^]**
:   the step size for optimizing the flexible constraints.
    Should be chosen as mu/(d^2^V/dq^2^)
    where mu is the reduced mass of two particles in a flexible constraint
    and d^2^V/dq^2^ is the second derivative of the potential
    in the constraint direction. Hopefully this number does not differ too
    much between the flexible constraints, as the number of iterations
    and thus the runtime is very sensitive to fcstep.
    Try several values!

  


---

### Test particle insertion

**rtpi: (0.05) [nm]**
:   the test particle insertion radius see integrators
    **tpi** and **tpic**

  


---

### Output control

**nstxout: (0) [steps]**
:   frequency to write coordinates to output
    trajectory file, the last coordinates are always written

**nstvout: (0) [steps]**
:   frequency to write velocities to output trajectory,
    the last velocities are always written

**nstfout: (0) [steps]**
:   frequency to write forces to output trajectory.

**nstlog: (1000) [steps]**
:   frequency to write energies to log file,
    the last energies are always written

**nstcalcenergy: (100)**
:   frequency for calculating the energies, 0 is never.
    This option is only relevant with dynamics.
    With a twin-range cut-off setup **nstcalcenergy** should be equal to
    or a multiple of **nstlist**.
    This option affects the performance in parallel simulations,
    because calculating energies requires global communication between all
    processes which can become a bottleneck at high parallelization.

**nstenergy: (1000) [steps]**
:   frequency to write energies to energy file,
    the last energies are always written,
    should be a multiple of **nstcalcenergy**.
    Note that the exact sums and fluctuations over all MD steps
    modulo **nstcalcenergy** are stored in the energy file,
    so g\_energy can report exact
    energy averages and fluctuations also when **nstenergy**>1

**nstxtcout: (0) [steps]**
:   frequency to write coordinates to xtc trajectory

**xtc-precision: (1000) [real]**
:   precision to write to xtc trajectory

**xtc-grps:**
:   group(s) to write to xtc trajectory, default the whole system is written
    (if **nstxtcout** > 0)

**energygrps:**
:   group(s) to write to energy file

  


---

### Neighbor searchingneighbor searching

**cutoff-scheme:**
:   **group**
    :   Generate a pair list for groups of atoms. These groups correspond to the
        charge groups in the topology. This was the only cut-off treatment scheme
        before version 4.6.
        There is no explicit buffering of the pair list. This enables efficient force
        calculations, but energy is only conserved when a buffer is explicitly added.
        For energy conservation, the **Verlet** option provides a more convenient
        and efficient algorithm.

    **Verlet**
    :   Generate a pair list with buffering. The buffer size is automatically set
        based on **verlet-buffer-drift**, unless this is set to -1, in which case
        **rlist** will be used. This option has an explicit, exact cut-off at
        **rvdw**=**rcoulomb**. Currently only cut-off, reaction-field,
        PME electrostatics and plain LJ are supported. Some mdrun functionality
        is not yet supported with the **Verlet** scheme, but grompp checks for this.
        Native GPU acceleration is only supported with **Verlet**. With GPU-accelerated PME,
        mdrun will automatically tune the CPU/GPU load balance by
        scaling **rcoulomb** and the grid spacing. This can be turned off with
        -notunepme.
        **Verlet** is somewhat faster than **group** when there is no water, or if **group** would use a pair-list buffer to conserve energy.

**nstlist: (10) [steps]**
:   **>0**
    :   Frequency to update the neighbor list (and
        the long-range forces, when using twin-range cut-offs). When this is 0,
        the neighbor list is made only once.
        With energy minimization the neighborlist will be updated for every
        energy evaluation when **nstlist**>0.
        With non-bonded force calculation on the GPU, a value of 20 or more gives
        the best performance.

    **0**
    :   The neighbor list is only constructed once and never updated.
        This is mainly useful for vacuum simulations in which all particles
        see each other.

    **-1**
    :   Automated update frequency, only supported with **cutoff-scheme**=**group**.
        This can only be used with switched, shifted or user potentials where
        the cut-off can be smaller than **rlist**. One then has a buffer
        of size **rlist** minus the longest cut-off.
        The neighbor list is only updated when one or more particles have moved further
        than half the buffer size from the center of geometry of their charge group
        as determined at the previous neighbor search.
        Coordinate scaling due to pressure coupling or the **deform** option
        is taken into account.
        This option guarantees that their are no cut-off artifacts,
        but for larger systems this can come at a high computational cost,
        since the neighbor list update frequency will be determined
        by just one or two particles moving slightly beyond the half buffer length
        (which does not necessarily imply that the neighbor list is invalid),
        while 99.99% of the particles are fine.

**nstcalclr: (-1) [steps]**
:   Controls the period between calculations of long-range forces when
    using the group cut-off scheme.

    **1**
    :   Calculate the long-range forces every single step. This is useful
        to have separate neighbor lists with buffers for electrostatics and Van
        der Waals interactions, and in particular it makes it possible to have
        the Van der Waals cutoff longer than electrostatics (useful e.g. with
        PME). However, there is no point in having identical long-range
        cutoffs for both interaction forms and update them every step - then
        it will be slightly faster to put everything in the short-range
        list.

    **>1**
    :   Calculate the long-range forces every **nstcalclr** steps and
        use a multiple-time-step integrator to combine forces. This can now be
        done more frequently than **nstlist** since the lists are stored,
        and it might be a good idea e.g. for Van der Waals interactions that
        vary slower than electrostatics.

    **-1**
    :   Calculate long-range forces on steps where neighbor searching is
        performed. While this is the default value, you might want to consider
        updating the long-range forces more frequently.

    Note that twin-range force evaluation might be enabled automatically
    by PP-PME load balancing. This is done in order to maintain the chosen
    Van der Waals interaction radius even if the load balancing is
    changing the electrostatics cutoff. If the .mdp file already
    specifies twin-range interactions (e.g. to evaluate Lennard-Jones
    interactions with a longer cutoff than the PME electrostatics every
    2-3 steps), the load balancing will have also a small effect on
    Lennard-Jones, since the short-range cutoff (inside which forces are
    evaluated every step) is changed.

**ns-type:**
:   **grid**
    :   Make a grid in the box and only check atoms in neighboring grid
        cells when constructing a new neighbor list every **nstlist** steps.
        In large systems grid search is much faster than simple search.

    **simple**
    :   Check every atom in the box when constructing a new neighbor list
        every **nstlist** steps.

**pbc:**
:   **xyz**
    :   Use periodic boundary conditions in all directions.

    **no**
    :   Use no periodic boundary conditions, ignore the box.
        To simulate without cut-offs, set all cut-offs to 0 and **nstlist**=0.
        For best performance without cut-offs, use **nstlist**=0,
        **ns-type**=simple
        and particle decomposition instead of domain decomposition.

    **xy**
    :   Use periodic boundary conditions in x and y directions only.
        This works only with **ns-type**=grid and can be used
        in combination with **walls**.
        Without walls or with only one wall the system size is infinite
        in the z direction. Therefore pressure coupling or Ewald summation
        methods can not be used.
        These disadvantages do not apply when two walls are used.

**periodic-molecules:**
:   **no**
    :   molecules are finite, fast molecular PBC can be used

    **yes**
    :   for systems with molecules that couple to themselves through
        the periodic boundary conditions, this requires a slower PBC algorithm
        and molecules are not made whole in the output

**verlet-buffer-drift: (0.005) [kJ/mol/ps]**
:   Useful only with **cutoff-scheme**=**Verlet**. This sets the target energy drift
    per particle caused by the Verlet buffer, which indirectly sets **rlist**.
    As both **nstlist** and the Verlet buffer size are fixed
    (for performance reasons), particle pairs not in the pair list can occasionally
    get within the cut-off distance during **nstlist**-1 nsteps. This
    generates energy drift. In a constant-temperature ensemble, the drift can be
    estimated for a given cut-off and **rlist**. The estimate assumes a
    homogeneous particle distribution, hence the drift might be slightly
    underestimated for multi-phase systems. For longer pair-list life-time
    (**nstlist**-1)\*dt the drift is overestimated, because the interactions
    between particles are ignored. Combined with cancellation of errors,
    the actual energy drift is usually one to two orders of magnitude smaller.
    Note that the generated buffer size takes into account that
    the GROMACS pair-list setup leads to a reduction in the drift by
    a factor 10, compared to a simple particle-pair based list.
    Without dynamics (energy minimization etc.), the buffer is 5% of the cut-off.
    For dynamics without temperature coupling or to override the buffer size,
    use **verlet-buffer-drift**=-1 and set **rlist** manually.

**rlist: (1) [nm]**
:   Cut-off distance for the short-range neighbor list.
    With **cutoff-scheme**=**Verlet**, this is by default set by the
    **verlet-buffer-drift** option and the value of **rlist** is ignored.

**rlistlong: (-1) [nm]**
:   Cut-off distance for the long-range neighbor list.
    This parameter is only relevant for a twin-range cut-off setup
    with switched potentials. In that case a buffer region is required to account
    for the size of charge groups. In all other cases this parameter
    is automatically set to the longest cut-off distance.

  


---

### Electrostaticselectrostatics

**coulombtype:**
:   **Cut-off**
    :   Twin range cut-offs with neighborlist cut-off **rlist** and
        Coulomb cut-off **rcoulomb**,
        where **rcoulomb**≥**rlist**.

        **Ewald**
        :   Classical Ewald sum electrostatics.
            The real-space cut-off **rcoulomb** should be equal to **rlist**.
            Use e.g. **rlist**=0.9, **rcoulomb**=0.9. The highest magnitude of
            wave vectors used in reciprocal space is controlled by **fourierspacing**.
            The relative accuracy of direct/reciprocal space
            is controlled by **ewald-rtol**.
              
            NOTE: Ewald scales as O(N^3/2^)
            and is thus extremely slow for large systems. It is included mainly for
            reference - in most cases PME will perform much better.

        **PME**
        :   Fast smooth Particle-Mesh Ewald (SPME) electrostatics. Direct space is similar
            to the Ewald sum, while the reciprocal part is performed with
            FFTs. Grid dimensions are controlled with **fourierspacing** and the
            interpolation order with **pme-order**. With a grid spacing of 0.1
            nm and cubic interpolation the electrostatic forces have an accuracy
            of 2-3\*10^-4^. Since the error from the vdw-cutoff is larger than this you
            might try 0.15 nm. When running in parallel the interpolation
            parallelizes better than the FFT, so try decreasing grid dimensions
            while increasing interpolation.

        **P3M-AD**
        :   Particle-Particle Particle-Mesh algorithm with analytical derivative
            for for long range electrostatic interactions. The method and code
            is identical to SPME, except that the influence function is optimized
            for the grid. This gives a slight increase in accuracy.

        **Reaction-Field electrostaticsreaction-field electrostatics**
        :   Reaction field with Coulomb cut-off **rcoulomb**,
            where **rcoulomb** ≥ **rlist**.
            The dielectric constant beyond the cut-off is **epsilon-rf**.
            The dielectric constant can be set to infinity by setting **epsilon-rf**=0.

        **Generalized-Reaction-Field**
        :   Generalized reaction field with Coulomb cut-off **rcoulomb**,
            where **rcoulomb** ≥ **rlist**.
            The dielectric constant beyond the cut-off is **epsilon-rf**.
            The ionic strength is computed from the number of charged
            (i.e. with non zero charge) charge groups.
            The temperature for the GRF potential is set with
            **ref-t** [K].

        **Reaction-Field-zero**
        :   In GROMACS, normal reaction-field electrostatics with
            **cutoff-scheme****=group** leads to bad
            energy conservation. **Reaction-Field-zero** solves this
            by making the potential zero beyond the cut-off. It can only
            be used with an infinite dielectric constant (**epsilon-rf=0**),
            because only for that value the force vanishes at the cut-off.
            **rlist** should be 0.1 to 0.3 nm larger than **rcoulomb**
            to accommodate for the size of charge groups and diffusion
            between neighbor list updates. This, and the fact that table lookups
            are used instead of analytical functions make **Reaction-Field-zero**
            computationally more expensive than normal reaction-field.

        **Reaction-Field-nec**
        :   The same as **Reaction-Field**, but implemented as in
            GROMACS versions before 3.3. No reaction-field correction is applied
            to excluded atom pairs and self pairs.
            The 1-4 interactions are calculated using a reaction-field.
            The missing correction due to the excluded pairs that do not have a 1-4
            interaction is up to a few percent of the total electrostatic
            energy and causes a minor difference in the forces and the pressure.

        **Shift**
        :   Analogous to **Shift** for **vdwtype**.
            You might want to use **Reaction-Field-zero** instead,
            which has a similar potential shape, but has a physical interpretation
            and has better energies due to the exclusion correction terms.

        **Encad-Shift**
        :   The Coulomb
            potential is decreased over the whole range, using the definition
            from the Encad simulation package.

        **Switch**
        :   Analogous to **Switch** for **vdwtype**.
            Switching the Coulomb potential can lead to serious artifacts,
            advice: use **Reaction-Field-zero** instead.

        **User**
        :   mdrun will now expect to find a file
            table.xvg with user-defined potential functions for
            repulsion, dispersion and Coulomb. When pair interactions are present,
            mdrun also expects to find a file tablep.xvg for
            the pair interactions. When the same interactions should be used
            for non-bonded and pair interactions the user can specify the same
            file name for both table files.
            These files should contain 7
            columns: the x value,
            f(x), -f'(x),
            g(x), -g'(x),
            h(x), -h'(x),
            where f(x) is the Coulomb function, g(x) the dispersion function
            and h(x) the repulsion function.
            When **vdwtype** is not set to **User** the values
            for g, -g', h and -h' are ignored.
            For the non-bonded interactions x values should run
            from 0 to the largest cut-off distance + **table-extension**
            and should be uniformly spaced. For the pair interactions the table
            length in the file will be used.
            The optimal spacing, which is used for non-user tables,
            is 0.002 [nm] when you run in single precision
            or 0.0005 [nm] when you run in double precision.
            The function value at x=0 is not important. More information is
            in the printed manual.

        **PME-Switch**
        :   A combination of PME and a switch function for the direct-space part
            (see above). **rcoulomb** is allowed to be smaller than **rlist**.
            This is mainly useful constant energy simulations (note that using
            **PME** with **cutoff-scheme**=**Verlet** will be more efficient).

        **PME-User**
        :   A combination of PME and user tables (see above).
            **rcoulomb** is allowed to be smaller than **rlist**.
            The PME mesh contribution is subtracted from the user table by mdrun.
            Because of this subtraction the user tables should contain
            about 10 decimal places.

        **PME-User-Switch**
        :   A combination of PME-User and a switching function (see above).
            The switching function is applied to final particle-particle interaction,
            i.e. both to the user supplied function and the PME Mesh correction part.

**coulomb-modifier:**
:   **Potential-shift-Verlet**
    :   Selects **Potential-shift** with the Verlet cutoff-scheme,
        as it is (nearly) free; selects **None** with the group cutoff-scheme.

    **Potential-shift**
    :   Shift the Coulomb potential by a constant such that it is zero at the cut-off.
        This makes the potential the integral of the force. Note that this does not
        affect the forces or the sampling.

    **None**
    :   Use an unmodified Coulomb potential.

**rcoulomb-switch: (0) [nm]**
:   where to start switching the Coulomb potential

**rcoulomb: (1) [nm]**
:   distance for the Coulomb cut-off

**epsilon-r: (1)**
:   The relative dielectric constant.
    A value of 0 means infinity.

**epsilon-rf: (0)**
:   The relative dielectric constant of the reaction field.
    This is only used with reaction-field electrostatics.
    A value of 0 means infinity.

---

### VdW

**vdwtype:**
:   **Cut-off**
    :   Twin range cut-offs with neighbor list cut-off **rlist** and
        VdW cut-off **rvdw**,
        where **rvdw** ≥ **rlist**.

    **Shift**
    :   The LJ (not Buckingham) potential is decreased over the whole
        range and the forces decay smoothly to zero between **rvdw-switch**
        and **rvdw**. The neighbor search cut-off **rlist** should be
        0.1 to 0.3 nm larger than **rvdw** to accommodate for the size of
        charge groups and diffusion between neighbor list
        updates.

    **Switch**
    :   The LJ (not Buckingham)
        potential is normal out to **rvdw-switch**, after which it is switched
        off to reach zero at **rvdw**. Both the potential and force functions
        are continuously smooth, but be aware that all switch functions will give rise
        to a bulge (increase) in the force (since we are switching the potential).
        The neighbor search cut-off **rlist** should be 0.1 to 0.3 nm larger than
        **rvdw** to accommodate for the size of charge groups and diffusion
        between neighbor list updates.

    **Encad-Shift**
    :   The LJ (not Buckingham)
        potential is decreased over the whole range, using the definition
        from the Encad simulation package.

    **User**
    :   See **user** for **coulombtype**.
        The function value at x=0 is not important. When you want to
        use LJ correction, make sure that **rvdw** corresponds to the
        cut-off in the user-defined function.
        When **coulombtype** is not set to **User** the values
        for f and -f' are ignored.

**vdw-modifier:**
:   **Potential-shift-Verlet**
    :   Selects **Potential-shift** with the Verlet cutoff-scheme,
        as it is (nearly) free; selects **None** with the group cutoff-scheme.

    **Potential-shift**
    :   Shift the Van der Waals potential by a constant such that it is zero at the cut-off.
        This makes the potential the integral of the force. Note that this does not
        affect the forces or the sampling.

    **None**
    :   Use an unmodified Van der Waals potential.

**rvdw-switch: (0) [nm]**
:   where to start switching the LJ potential

**rvdw: (1) [nm]**
:   distance for the LJ or Buckingham cut-off

**DispCorr:**


**no**
:   don't apply any correction

**EnerPres**
:   apply long range dispersion corrections for Energy
    and Pressure

**Ener**
:   apply long range dispersion corrections for Energy
    only


---

### Tables

**table-extension: (1) [nm]**
:   Extension of the non-bonded potential lookup tables beyond the largest cut-off distance.
    The value should be large enough to account for charge group sizes
    and the diffusion between neighbor-list updates.
    Without user defined potential the same table length is used
    for the lookup tables for the 1-4 interactions,
    which are always tabulated irrespective of the use of
    tables for the non-bonded interactions.

**energygrp-table:**
:   When user tables are used for electrostatics and/or VdW,
    here one can give pairs of energy groups for which seperate
    user tables should be used.
    The two energy groups will be appended to the table file name,
    in order of their definition in **energygrps**, seperated by underscores.
    For example, if energygrps = Na Cl Sol
    and energygrp-table = Na Na Na Cl, mdrun will read
    table\_Na\_Na.xvg and table\_Na\_Cl.xvg in addition
    to the normal table.xvg which will be used for all other
    energy group pairs.

---

### Ewald

**fourierspacing: (0.12) [nm]**
:   For ordinary Ewald, the ratio of the box dimensions and the spacing
    determines a lower bound for the number of wave vectors to use in each
    (signed) direction. For PME and P3M, that ratio determines a lower bound
    for the number of Fourier-space grid points that will be used along that
    axis. In all cases, the number for each direction can be overridden by
    entering a non-zero value for **fourier\_n[xyz]**.
    For optimizing the relative load of the particle-particle interactions
    and the mesh part of PME, it is useful to know that
    the accuracy of the electrostatics remains nearly constant
    when the Coulomb cut-off and the PME grid spacing are scaled
    by the same factor.

**fourier-nx (0) ; fourier-ny (0) ; fourier-nz: (0)**
:   Highest magnitude of wave vectors in reciprocal space when using Ewald.
:   Grid size when using PME or P3M. These values override
    **fourierspacing** per direction. The best choice is powers of
    2, 3, 5 and 7. Avoid large primes.

**pme-order (4)**
:   Interpolation order for PME. 4 equals cubic interpolation. You might try
    6/8/10 when running in parallel and simultaneously decrease grid dimension.

**ewald-rtol (1e-5)**
:   The relative strength of the Ewald-shifted direct potential at
    **rcoulomb** is given by **ewald-rtol**.
    Decreasing this will give a more accurate direct sum,
    but then you need more wave vectors for the reciprocal sum.

**ewald-geometry: (3d)**
:   **3d**
    :   The Ewald sum is performed in all three dimensions.

    **3dc**
    :   The reciprocal sum is still performed in 3D,
        but a force and potential correction applied in the z
        dimension to produce a pseudo-2D summation.
        If your system has a slab geometry in the x-y plane you can
        try to increase the z-dimension of the box (a box height of 3 times
        the slab height is usually ok)
        and use this option.

**epsilon-surface: (0)**
:   This controls the dipole correction to the Ewald summation in 3D. The
    default value of zero means it is turned off. Turn it on by setting it to the value
    of the relative permittivity of the imaginary surface around your infinite system. Be
    careful - you shouldn't use this if you have free mobile charges in your system.
    This value does not affect the slab 3DC variant of the long range corrections.

**optimize-fft:**
:   **no**
    :   Don't calculate the optimal FFT plan for the grid at startup.

    **yes**
    :   Calculate the optimal FFT plan for the grid at startup. This saves a
        few percent for long simulations, but takes a couple of minutes
        at start.

  


---

### Temperature couplingtemperature coupling

**tcoupl:**
:   **no**
    :   No temperature coupling.

    **berendsen**
    :   Temperature coupling with a Berendsen-thermostat to a bath with
        temperature **ref-t** [K], with time constant **tau-t** [ps].
        Several groups can be coupled separately, these are specified in the
        **tc-grps** field separated by spaces.

    **nose-hoover**
    :   Temperature coupling using a Nose-Hoover extended
        ensemble. The reference temperature and coupling groups are selected
        as above, but in this case **tau-t** [ps] controls the period
        of the temperature fluctuations at equilibrium, which is slightly
        different from a relaxation time.
        For NVT simulations the conserved energy quantity is written
        to energy and log file.

    **v-rescale**
    :   Temperature coupling using velocity rescaling with a stochastic term
        (JCP 126, 014101).
        This thermostat is similar to Berendsen coupling, with the same scaling
        using **tau-t**, but the stochastic term ensures that a proper
        canonical ensemble is generated. The random seed is set with
        **ld-seed**.
        This thermostat works correctly even for **tau-t**=0.
        For NVT simulations the conserved energy quantity is written
        to the energy and log file.

    **nsttcouple: (-1)**
    :   The frequency for coupling the temperature.
        The default value of -1 sets **nsttcouple** equal to **nstlist**,
        unless **nstlist**≤0, then a value of 10 is used.
        For velocity Verlet integrators **nsttcouple** is set to 1.

**nh-chain-length (10)**
:   the number of chained Nose-Hoover thermostats for velocity Verlet integrators, the leap-frog **md** integrator only supports 1. Data for the NH chain variables is not printed to the .edr, but can be using the GMX\_NOSEHOOVER\_CHAINS environment variable

**tc-grps:**
:   groups to couple separately to temperature bath

**tau-t: [ps]**
:   time constant for coupling (one for each group in **tc-grps**),
    -1 means no temperature coupling

**ref-t: [K]**
:   reference temperature for coupling (one for each group in **tc-grps**)

  


---

### Pressure couplingpressure coupling

**pcoupl:**
:   **no**
    :   No pressure coupling. This means a fixed box size.

    **berendsen**
    :   Exponential relaxation pressure coupling with time constant
        **tau-p** [ps]. The box is scaled every timestep. It has been
        argued that this does not yield a correct thermodynamic ensemble,
        but it is the most efficient way to scale a box at the beginning
        of a run.

    **Parrinello-Rahman**
    :   Extended-ensemble pressure coupling where the box vectors are
        subject to an equation of motion. The equation of motion for the atoms
        is coupled to this. No instantaneous scaling takes place. As for
        Nose-Hoover temperature coupling the time constant **tau-p** [ps]
        is the period of pressure fluctuations at equilibrium. This is
        probably a better method when you want to apply pressure scaling
        during data collection, but beware that you can get very large
        oscillations if you are starting from a different pressure. For
        simulations where the exact fluctation of the NPT ensemble are
        important, or if the pressure coupling time is very short it may not
        be appropriate, as the previous time step pressure is used in some
        steps of the GROMACS implementation for the current time step pressure.

**MTTK**
:   Martyna-Tuckerman-Tobias-Klein implementation, only useable with **md-vv**
    or **md-vv-avek**, very similar to Parrinello-Rahman.
    As for Nose-Hoover temperature coupling the time constant **tau-p**
    [ps] is the period of pressure fluctuations at equilibrium. This is
    probably a better method when you want to apply pressure scaling
    during data collection, but beware that you can get very large
    oscillations if you are starting from a different pressure. Currently only supports isotropic scaling.

**pcoupltype:**
:   **isotropic**
    :   Isotropic pressure coupling with time constant **tau-p** [ps].
        The compressibility and reference pressure are set with
        **compressibility** [bar^-1^] and **ref-p** [bar], one
        value is needed.

    **semiisotropic**
    :   Pressure coupling which is isotropic in the x and y direction,
        but different in the z direction.
        This can be useful for membrane simulations.
        2 values are needed for x/y and z directions respectively.

    **anisotropic**
    :   Idem, but 6 values are needed for xx, yy, zz, xy/yx, xz/zx and yz/zy
        components, respectively.
        When the off-diagonal compressibilities are set to zero,
        a rectangular box will stay rectangular.
        Beware that anisotropic scaling can lead to extreme deformation
        of the simulation box.

    **surface-tension**
    :   Surface tension coupling for surfaces parallel to the xy-plane.
        Uses normal pressure coupling for the z-direction, while the surface tension
        is coupled to the x/y dimensions of the box.
        The first **ref-p** value is the reference surface tension times
        the number of surfaces [bar nm],
        the second value is the reference z-pressure [bar].
        The two **compressibility** [bar^-1^] values are the compressibility
        in the x/y and z direction respectively.
        The value for the z-compressibility should be reasonably accurate since it
        influences the convergence of the surface-tension, it can also be set to zero
        to have a box with constant height.

**nstpcouple: (-1)**
:   The frequency for coupling the pressure.
    The default value of -1 sets **nstpcouple** equal to **nstlist**,
    unless **nstlist** ≤0, then a value of 10 is used.
    For velocity Verlet integrators **nstpcouple** is set to 1.

**tau-p: (1) [ps]**
:   time constant for coupling

**compressibility: [bar^-1^]**
:   compressibility (NOTE: this is now really in bar^-1^)
    For water at 1 atm and 300 K the compressibility is 4.5e-5 [bar^-1^].

**ref-p: [bar]**
:   reference pressure for coupling

**refcoord-scaling:**
:   **no**
    :   The reference coordinates for position restraints are not modified.
        Note that with this option the virial and pressure will depend on the absolute
        positions of the reference coordinates.

    **all**
    :   The reference coordinates are scaled with the scaling matrix of the pressure coupling.

    **com**
    :   Scale the center of mass of the reference coordinates with the scaling matrix of the pressure coupling. The vectors of each reference coordinate to the center of mass are not scaled. Only one COM is used, even when there are multiple molecules with position restraints. For calculating the COM of the reference coordinates in the starting configuration, periodic boundary conditions are not taken into account.

  


---

### Simulated annealingsimulated annealing

Simulated annealing is controlled separately for each temperature group in GROMACS. The reference temperature is a piecewise linear function, but you can use an arbitrary number of points for each group, and choose either a single sequence or a periodic behaviour for each group. The actual annealing is performed by dynamically changing the reference temperature used in the thermostat algorithm selected, so remember that the system will usually not instantaneously reach the reference temperature!

**annealing:**
:   Type of annealing for each temperature group


**no**
:   No simulated annealing - just couple to reference temperature value.

**single**
:   A single sequence of annealing points. If your simulation is longer than the time of the last point, the temperature will be coupled to this constant value after the annealing sequence has reached the last time point.

**periodic**
:   The annealing will start over at the first reference point once the last reference time is reached. This is repeated until the simulation ends.

**annealing-npoints:**
:   A list with the number of annealing reference/control points used for
    each temperature group. Use 0 for groups that are not annealed. The number of entries should equal the number of temperature groups.

**annealing-time:**
:   List of times at the annealing reference/control points for each group. If you are using periodic annealing, the times will be used modulo the last value, i.e. if the values are 0, 5, 10, and 15, the coupling will restart at the 0ps value after 15ps, 30ps, 45ps, etc. The number of entries should equal the sum of the numbers given in annealing-npoints.

**annealing-temp:**
:   List of temperatures at the annealing reference/control points for each group. The number of entries should equal the sum of the numbers given in annealing-npoints.
  
Confused? OK, let's use an example. Assume you have two temperature groups, set the group selections to annealing = single periodic, the number of points of each group to annealing-npoints = 3 4, the times to annealing-time = 0 3 6 0 2 4 6 and finally temperatures to annealing-temp = 298 280 270 298 320 320 298.
The first group will be coupled to 298K at 0ps, but the reference temperature will drop linearly to reach 280K at 3ps, and then linearly between 280K and 270K from 3ps to 6ps. After this is stays constant, at 270K. The second group is coupled to 298K at 0ps, it increases linearly to 320K at 2ps, where it stays constant until 4ps. Between 4ps and 6ps it decreases to 298K, and then it starts over with the same pattern again, i.e. rising linearly from 298K to 320K between 6ps and 8ps. Check the summary printed by grompp if you are unsure!
  


---

### Velocity generation

**gen-vel:**
:   **no**
    :   Do not generate velocities. The velocities are set to zero
        when there are no velocities in the input structure file.

    **yes**
    :   Generate velocities in grompp according to a Maxwell distribution at
        temperature **gen-temp** [K], with random seed **gen-seed**.
        This is only meaningful with integrator **md**.

**gen-temp: (300) [K]**
:   temperature for Maxwell distribution

**gen-seed: (173529) [integer]**
:   used to initialize random generator for random velocities,
    when **gen-seed** is set to -1, the seed is calculated from
    the process ID number.

  


---

### Bonds

**constraintsconstraint algorithms:**
:   **none**
    :   No constraints except for those defined explicitly in the topology,
        i.e. bonds are represented by a harmonic (or other) potential
        or a Morse potential (depending on the setting of **morse**)
        and angles by a harmonic (or other) potential.

        **h-bonds**
        :   Convert the bonds with H-atoms to constraints.

        **all-bonds**
        :   Convert all bonds to constraints.

        **h-angles**
        :   Convert all bonds and additionally the angles that involve H-atoms
            to bond-constraints.

        **all-angles**
        :   Convert all bonds and angles to bond-constraints.

    **constraint-algorithm:**
    :   **LINCS**
        :   LINear Constraint Solver.
            With domain decomposition the parallel version P-LINCS is used.
            The accuracy in set with
            **lincs-order**, which sets the number of matrices in the expansion
            for the matrix inversion.
            After the matrix inversion correction the algorithm does
            an iterative correction to compensate for lengthening due to rotation.
            The number of such iterations can be controlled with
            **lincs-iter**. The root mean square relative constraint deviation
            is printed to the log file every **nstlog** steps.
            If a bond rotates more than **lincs-warnangle** [degrees] in one step,
            a warning will be printed both to the log file and to stderr.
            LINCS should not be used with coupled angle constraints.

        **SHAKE**
        :   SHAKE is slightly slower and less stable than LINCS, but does work with
            angle constraints.
            The relative tolerance is set with **shake-tol**, 0.0001 is a good value
            for ``normal'' MD. SHAKE does not support constraints between atoms
            on different nodes, thus it can not be used with domain decompositon
            when inter charge-group constraints are present.
            SHAKE can not be used with energy minimization.

    **continuation:**
    :   This option was formerly known as unconstrained-start.
    :   **no**
        :   apply constraints to the start configuration and reset shells

        **yes**
        :   do not apply constraints to the start configuration
            and do not reset shells, useful for exact coninuation and reruns

    **shake-tol: (0.0001)**
    :   relative tolerance for SHAKE

    **lincs-order: (4)**
    :   Highest order in the expansion of the constraint coupling matrix.
        When constraints form triangles, an additional expansion of the same
        order is applied on top of the normal expansion only for the couplings
        within such triangles.
        For ``normal'' MD simulations an order of 4 usually suffices, 6 is
        needed for large time-steps with virtual sites or BD.
        For accurate energy minimization an order of 8 or more might be required.
        With domain decomposition, the cell size is limited by the distance
        spanned by **lincs-order**+1 constraints. When one wants to scale
        further than this limit, one can decrease **lincs-order** and increase
        **lincs-iter**, since the accuracy does not deteriorate
        when (1+**lincs-iter**)\***lincs-order** remains constant.

    **lincs-iter: (1)**
    :   Number of iterations to correct for rotational lengthening in LINCS.
        For normal runs a single step is sufficient, but for NVE
        runs where you want to conserve energy accurately or for accurate
        energy minimization you might want to increase it to 2.

        **lincs-warnangle:** (30) [degrees]
        :   maximum angle that a bond can rotate before LINCS will complain

        **morse:**
        :   **no**
            :   bonds are represented by a harmonic potential

            **yes**
            :   bonds are represented by a Morse potential

  


---

### Energy group exclusions

**energygrp-excl:**
:   Pairs of energy groups for which all non-bonded interactions are
    excluded. An example: if you have two energy groups Protein
    and SOL, specifying
      
    energygrp-excl = Protein Protein  SOL SOL
      
    would give only the non-bonded interactions between the protein and the
    solvent. This is especially useful for speeding up energy calculations with
    mdrun -rerun and for excluding interactions within frozen groups.

  


---

### Wallswalls

**nwall: 0**
:   When set to **1** there is a wall at z=0, when set to **2**
    there is also a wall at z=z-box. Walls can only be used with **pbc=xy**.
    When set to **2** pressure coupling and Ewald summation can be used
    (it is usually best to use semiisotropic pressure coupling with
    the x/y compressibility set to 0, as otherwise the surface area will change).
    Walls interact wit the rest of the system through an optional wall-atomtype.
    Energy groups wall0 and wall1 (for **nwall=2**) are
    added automatically to monitor the interaction of energy groups
    with each wall.
    The center of mass motion removal will be turned
    off in the z-direction.

**wall-atomtype:**
:   the atom type name in the force field for each wall.
    By (for example) defining a special wall atom type in the topology with its
    own combination rules, this allows for independent tuning of the interaction
    of each atomtype with the walls.

**wall-type:**
:   **9-3**
    :   LJ integrated over the volume behind the wall: 9-3 potential

    **10-4**
    :   LJ integrated over the wall surface: 10-4 potential

    **12-6**
    :   direct LJ potential with the z distance from the wall

    **table**
    :   user defined potentials indexed with the z distance from the wall, the tables are read analogously to
        the **energygrp-table** option,
        where the first name is for a ``normal'' energy group and the second name
        is wall0 or wall1,
        only the dispersion and repulsion columns are used

**wall-r-linpot: -1 (nm)**
:   Below this distance from the wall the potential is continued
    linearly and thus the force is constant. Setting this option to
    a postive value is especially useful for equilibration when some atoms
    are beyond a wall.
    When the value is ≤0 (<0 for **wall-type=table**),
    a fatal error is generated when atoms are beyond a wall.

**wall-density: [nm^-3^/nm^-2^]**
:   the number density of the atoms for each wall for wall types
    **9-3** and **10-4**

    **wall-ewald-zfac: 3**
    :   The scaling factor for the third box vector for Ewald summation only,
        the minimum is 2.
        Ewald summation can only be used with **nwall=2**, where one
        should use **ewald-geometry=3dc**.
        The empty layer in the box serves to decrease the unphysical Coulomb
        interaction between periodic images.

  


---

### COM pulling

**pull:**
:   **no**
    :   No center of mass pulling.
        All the following pull options will be ignored
        (and if present in the .mdp file, they unfortunately generate warnings)

    **umbrella**
    :   Center of mass pulling using an umbrella potential
        between the reference group and one or more groups.

    **constraint**
    :   Center of mass pulling using a constraint
        between the reference group and one or more groups.
        The setup is identical to the option **umbrella**, except for the fact
        that a rigid constraint is applied instead of a harmonic potential.

    **constant-force**
    :   Center of mass pulling using a linear potential and therefore
        a constant force. For this option there is no reference position
        and therefore the parameters **pull-init** and **pull-rate**
        are not used.

**pull-geometry:**
:   **distance**
    :   Pull along the vector connecting the two groups.
        Components can be selected with **pull-dim**.

    **direction**
    :   Pull in the direction of **pull-vec**.

    **direction-periodic**
    :   As **direction**, but allows the distance to be larger than
        half the box size. With this geometry the box should not be dynamic
        (e.g. no pressure scaling) in the pull dimensions and the pull force
        is not added to virial.

    **cylinder**
    :   Designed for pulling with respect to a layer where the reference COM
        is given by a local cylindrical part of the reference group.
        The pulling is in the direction of **pull-vec**.
        From the reference group a cylinder is selected around the axis going
        through the pull group with direction **pull-vec** using two radii.
        The radius **pull-r1** gives the radius within which all
        the relative weights are one, between **pull-r1** and
        **pull-r0** the weights are switched to zero. Mass weighting is also used.
        Note that the radii should be smaller than half the box size.
        For tilted cylinders they should be even smaller than half the box size
        since the distance of an atom in the reference group
        from the COM of the pull group has both a radial and an axial component.

        **position**
        :   Pull to the position of the reference group plus
            **pull-init** + time\***pull-rate**\***pull-vec**.

**pull-dim: (Y Y Y)**
:   the distance components to be used with geometry **distance**
    and **position**, and also sets which components are printed
    to the output files

**pull-r1: (1) [nm]**
:   the inner radius of the cylinder for geometry **cylinder**

**pull-r0: (1) [nm]**
:   the outer radius of the cylinder for geometry **cylinder**

**pull-constr-tol: (1e-6)**
:   the relative constraint tolerance for constraint pulling

**pull-start:**
:   **no**
    :   do not modify **pull-init**

        **yes**
        :   add the COM distance of the starting conformation to **pull-init**

    **pull-nstxout: (10)**
    :   frequency for writing out the COMs of all the pull group

    **pull-nstfout: (1)**
    :   frequency for writing out the force of all the pulled group

    **pull-ngroups: (1)**
    :   The number of pull groups, not including the reference group.
        If there is only one group, there is no difference in treatment
        of the reference and pulled group (except with the cylinder geometry).
        Below only the pull options for the reference group (ending on 0)
        and the first group (ending on 1) are given,
        further groups work analogously, but with the number 1 replaced
        by the group number.

    **pull-group0:**
    :   The name of the reference group. When this is empty an absolute reference
        of (0,0,0) is used. With an absolute reference the system is no longer
        translation invariant and one should think about what to do with
        the center of mass motion.

    **pull-weights0:**
    :   see **pull-weights1**

    **pull-pbcatom0: (0)**
    :   see **pull-pbcatom1**

    **pull-group1:**
    :   The name of the pull group.

    **pull-weights1:**
    :   Optional relative weights which are multiplied with the masses of the atoms
        to give the total weight for the COM. The number should be 0, meaning all 1,
        or the number of atoms in the pull group.

    **pull-pbcatom1: (0)**
    :   The reference atom for the treatment of periodic boundary conditions
        inside the group
        (this has no effect on the treatment of the pbc between groups).
        This option is only important when the diameter of the pull group
        is larger than half the shortest box vector.
        For determining the COM, all atoms in the group are put at their periodic image
        which is closest to **pull-pbcatom1**.
        A value of 0 means that the middle atom (number wise) is used.
        This parameter is not used with geometry **cylinder**.
        A value of -1 turns on cosine weighting, which is useful for a group
        of molecules in a periodic system, e.g. a water slab (see Engin et al.
        J. Chem. Phys. B 2010).

    **pull-vec1: (0.0 0.0 0.0)**
    :   The pull direction. grompp normalizes the vector.

    **pull-init1: (0.0) / (0.0 0.0 0.0) [nm]**
    :   The reference distance at t=0. This is a single value,
        except for geometry **position** which uses a vector.

    **pull-rate1: (0) [nm/ps]**
    :   The rate of change of the reference position.

    **pull-k1: (0) [kJ mol^-1^ nm^-2^] / [kJ mol^-1^ nm^-1^]**
    :   The force constant. For umbrella pulling this is the harmonic force
        constant in [kJ mol^-1^ nm^-2^]. For constant force pulling
        this is the force constant of the linear potential, and thus minus (!)
        the constant force in [kJ mol^-1^ nm^-1^].

    **pull-kB1: (pull-k1) [kJ mol^-1^ nm^-2^] / [kJ mol^-1^ nm^-1^]**
    :   As **pull-k1**, but for state B. This is only used when
        **free-energy** is turned on.
        The force constant is then (1 - lambda)\***pull-k1** + lambda\***pull-kB1**.

  


---

### NMR refinement

**disre:**
:   **no**
    :   ignore distance restraint information in topology file

    **simple**
    :   simple (per-molecule) distance restraints.

        **ensemble**
        :   distance restraints over an ensemble of molecules in one
            simulation box. Normally, one would perform ensemble averaging over
            multiple subsystems, each in a separate box, using mdrun -multi;s
            upply topol0.tpr, topol1.tpr, ... with different
            coordinates and/or velocities.
            The environment variable GMX\_DISRE\_ENSEMBLE\_SIZE sets the number
            of systems within each ensemble (usually equal to the mdrun -multi value).

**disre-weighting:**
:   **equal** (default)
    :   divide the restraint force equally over all atom pairs in the restraint

    **conservative**
    :   the forces are the derivative of the restraint potential,
        this results in an r^-7^ weighting of the atom pairs.
        The forces are conservative when disre-tau is zero.

**disre-mixed:**
:   **no**
    :   the violation used in the calculation of the restraint force is the
        time-averaged violation

    **yes**
    :   the violation used in the calculation of the restraint force is the
        square root of the product of the time-averaged violation and the instantaneous violation

**disre-fc: (1000) [kJ mol^-1^ nm^-2^]**
:   force constant for distance restraints, which is multiplied by a
    (possibly) different factor for each restraint given in the fac
    column of the interaction in the topology file.

**disre-tau: (0) [ps]**
:   time constant for distance restraints running average. A value of zero turns off time averaging.

**nstdisreout: (100) [steps]**
:   period between steps when the running time-averaged and instantaneous distances
    of all atom pairs involved in restraints are written to the energy file
    (can make the energy file very large)

**orire:**
:   **no**
    :   ignore orientation restraint information in topology file

    **yes**
    :   use orientation restraints, ensemble averaging can be performed
        with mdrun -multi

    **orire-fc: (0) [kJ mol]**
    :   force constant for orientation restraints, which is multiplied by a
        (possibly) different weight factor for each restraint, can be set to zero to
        obtain the orientations from a free simulation

    **orire-tau: (0) [ps]**
    :   time constant for orientation restraints running average. A value of zero turns off time averaging.

    **orire-fitgrp:**
    :   fit group for orientation restraining. This group of atoms is used
        to determine the rotation **R** of the system with respect to the
        reference orientation. The reference orientation is the starting
        conformation of the first subsystem. For a protein, backbone is a reasonable
        choice

    **nstorireout: (100) [steps]**
    :   period between steps when the running time-averaged and instantaneous orientations
        for all restraints, and the molecular order tensor are written to the energy file
        (can make the energy file very large)

  


---

### Free energy calculationsfree energy calculations

**free-energy:**
:   **no**
    :   Only use topology A.

    **yes**
    :   Interpolate between topology A (lambda=0) to topology B (lambda=1)
        and write the derivative of the Hamiltonian with respect to lambda (as specified with **dhdl-derivatives**), or the Hamiltonian differences with respect to other lambda values (as specified with **foreign-lambda**) to
        the energy file and/or to dhdl.xvg, where they can be processed by, for example g\_bar.
        The potentials, bond-lengths and angles are interpolated linearly as
        described in the manual. When **sc-alpha** is larger than zero, soft-core
        potentials are used for the LJ and Coulomb interactions.

    **expanded**
    :   Turns on expanded ensemble simulation, where the alchemical state becomes a dynamic variable, allowing jumping between different Hamiltonians. See the expanded ensemble options for controlling how expanded ensemble simulations are performed. The different Hamiltonians used in expanded ensemble simulations are defined by the other free energy options.

**init-lambda: (-1)**
:   starting value for lambda (float). Generally, this should only be used with slow growth (i.e. nonzero **delta-lambda**). In other cases, **init-lambda-state** should be specified instead. Must be greater than or equal to 0.

**delta-lambda: (0)**
:   increment per time step for lambda

**init-lambda-state: (-1)**
:   starting value for the lambda state (integer). Specifies which columm of the lambda vector (**coul-lambdas**, **vdw-lambdas**, **bonded-lambdas**, **restraint-lambdas**, **mass-lambdas**, **temperature-lambdas**, **fep-lambdas**) should be used. This is a zero-based index: **init-lambda-state** 0 means the first column, and so on.

**fep-lambdas: ()**
:   Zero, one or more lambda values for which Delta H values will
    be determined and written to dhdl.xvg every **nstdhdl** steps.
    Values must be between 0 and 1.
    Free energy differences between different lambda values can then
    be determined with g\_bar. **fep-lambdas** is different from the other -lambdas keywords because
    all components of the lambda vector that are not specified will use **fep-lambdas** (including restraint-lambdas and therefore the pull code restraints).

**coul-lambdas: ()**
:   Zero, one or more lambda values for which Delta H values will
    be determined and written to dhdl.xvg every **nstdhdl** steps. Values must be between 0 and 1.
    Only the electrostatic interactions are controlled with this component of the lambda vector (and only if the lambda=0 and lambda=1 states have differing electrostatic interactions).

**vdw-lambdas: ()**
:   Zero, one or more lambda values for which Delta H values will
    be determined and written to dhdl.xvg every **nstdhdl** steps. Values must be between 0 and 1.
    Only the van der Waals interactions are controlled with this component of the lambda vector.

**bonded-lambdas: ()**
:   Zero, one or more lambda values for which Delta H values will
    be determined and written to dhdl.xvg every **nstdhdl** steps. Values must be between 0 and 1.
    Only the bonded interactions are controlled with this component of the lambda vector.

**restraint-lambdas: ()**
:   Zero, one or more lambda values for which Delta H values will
    be determined and written to dhdl.xvg every **nstdhdl** steps. Values must be between 0 and 1.
    Only the restraint interactions: dihedral restraints, and the pull code restraints are controlled with this component of the lambda vector.

**mass-lambdas: ()**
:   Zero, one or more lambda values for which Delta H values will
    be determined and written to dhdl.xvg every **nstdhdl** steps. Values must be between 0 and 1.
    Only the particle masses are controlled with this component of the lambda vector.

**temperature-lambdas: ()**
:   Zero, one or more lambda values for which Delta H values will
    be determined and written to dhdl.xvg every **nstdhdl** steps. Values must be between 0 and 1.
    Only the temperatures controlled with this component of the lambda vector.
    Note that these lambdas should not be used for replica exchange, only for simulated tempering.

**calc-lambda-neighbors (1)**
:   Controls the number of lambda values for which Delta H values will be
    calculated and written out, if **init-lambda-state** has been set. A
    positive value will limit the number of lambda points calculated to only the
    nth neighbors of **init-lambda-state**: for example, if
    **init-lambda-state** is 5 and this parameter has a value of 2, energies for
    lambda points 3-7 will be calculated and writen out. A value of -1 means all
    lambda points will be written out. For normal BAR such as with g\_bar, a value
    of 1 is sufficient, while for MBAR -1 should be used.

**sc-alpha: (0)**
:   the soft-core alpha parameter, a value of 0 results in linear interpolation of the LJ and Coulomb interactions

**sc-r-power: (6)**
:   the power of the radial term in the soft-core equation. Possible values are 6 and 48. 6 is more standard, and is the default. When 48 is used, then sc-alpha should generally be much lower (between 0.001 and 0.003).

**sc-coul: (no)**
:   Whether to apply the soft core free energy interations to the Columbic interaction. Default is no, as it is generally
    more efficient to turn of the Coulomic interactions linearly before turning off electrostatic interactions.

**sc-power: (0)**
:   the power for lambda in the soft-core function, only the values 1 and 2 are supported

**sc-sigma: (0.3) [nm]**
:   the soft-core sigma for particles which have a C6 or C12 parameter equal
    to zero or a sigma smaller than **sc-sigma**

**couple-moltype:**
:   Here one can supply a molecule type (as defined in the topology)
    for calculating solvation or coupling free energies.
    There is a special option **system** that couples all molecule types
    in the system. This can be useful for equilibrating a system
    starting from (nearly) random coordinates.
    **free-energy** has to be turned on.
    The Van der Waals interactions and/or charges in this molecule type can be
    turned on or off between lambda=0 and lambda=1, depending on the settings
    of **couple-lambda0** and **couple-lambda1**. If you want to decouple
    one of several copies of a molecule, you need to copy and rename
    the molecule definition in the topology.

**couple-lambda0:**
:   **vdw-q**
    :   all interactions are on at lambda=0

        **vdw**
        :   the charges are zero (no Coulomb interactions) at lambda=0

            **q**
            :   the Van der Waals interactions are turned at lambda=0; soft-core interactions will be required to avoid singularities

                **none**
                :   the Van der Waals interactions are turned off and the charges are zero at lambda=0; soft-core interactions will be required to avoid singularities.

    **couple-lambda1:**
    :   analogous to **couple-lambda1**, but for lambda=1

        **couple-intramol:**
        :   **no**
            :   All intra-molecular non-bonded interactions for moleculetype **couple-moltype** are replaced by exclusions and explicit pair interactions. In this manner the decoupled state of the molecule corresponds to the proper vacuum state without periodicity effects.

                **yes**
                :   The intra-molecular Van der Waals and Coulomb interactions are also turned on/off. This can be useful for partitioning free-energies of relatively large molecules, where the intra-molecular non-bonded interactions might lead to kinetically trapped vacuum conformations. The 1-4 pair interactions are not turned off.

            **nstdhdl: (100)**
            :   the frequency for writing dH/dlambda and possibly Delta H to dhdl.xvg,
                0 means no ouput, should be a multiple of **nstcalcenergy**
            .

        **dhdl-derivatives: (yes)**
        :   If yes (the default), the derivatives of the Hamiltonian with respect to lambda at each **nstdhdl** step are written out. These values are needed for interpolation of linear energy differences with g\_bar (although the same can also be achieved with the right **foreign lambda** setting, that may not be as flexible), or with thermodynamic integration

        **dhdl-print-energy: (no)**
        :   Include the total energy in the dhdl file. This information is needed for later analysis if the states of interest in the free e energy calculation are at different temperatures. If all are at the same temperature, this information is not needed.

        **separate-dhdl-file: (yes)**
        :   **yes**
            :   the free energy values that are calculated (as specified with the **foreign-lambda** and **dhdl-derivatives** settings) are written out to a separate file, with the default name dhdl.xvg. This file can be used directly with g\_bar.

            **no**
            :   The free energy values are written out to the energy output file (ener.edr, in accumulated blocks at every **nstenergy** steps), where they can be extracted with g\_energy or used directly with g\_bar.

            **dh-hist-size: (0)**
            :   If nonzero, specifies the size of the histogram into which the Delta H values (specified with **foreign-lambda**) and the derivative dH/dl values are binned, and written to ener.edr. This can be used to save disk space while calculating free energy differences. One histogram gets written for each **foreign lambda** and two for the dH/dl, at every **nstenergy** step. Be aware that incorrect histogram settings (too small size or too wide bins) can introduce errors. Do not use histograms unless you're certain you need it.

            **dh-hist-spacing (0.1)**
            :   Specifies the bin width of the histograms, in energy units. Used in conjunction with **dh-hist-size**. This size limits the accuracy with which free energies can be calculated. Do not use histograms unless you're certain you need it.

  


---

### Expanded Ensemble calculations

**nstexpanded**
:   The number of integration steps beween attempted moves changing the system Hamiltonian in expanded ensemble simulations. Must be a multiple of **nstcalcenergy**, but can be greater or less than **nstdhdl**.

**lmc-stats:**
:   **no**
    :   No Monte Carlo in state space is performed.

    **metropolis-transition**
    :   Uses the Metropolis weights to update the expanded ensemble weight of each state.
        Min{1,exp(-(beta\_new u\_new - beta\_old u\_old)}

    **barker-transition**
    :   Uses the Barker transition critera to update the expanded ensemble weight of each state i, defined by
        exp(-beta\_new u\_new)/[exp(-beta\_new u\_new)+exp(-beta\_old u\_old)

    **wang-landau**
    :   Uses the Wang-Landau algorithm (in state space, not energy space) to update the expanded ensemble weights.

    **min-variance**
    :   Uses the minimum variance updating method of Escobedo et al. to update the expanded ensemble weights. Weights
        will not be the free energies, but will rather emphasize states that need more sampling to give even uncertainty.

    **lmc-mc-move:**
    :   **no**
        :   No Monte Carlo in state space is performed.

        **metropolis-transition**
        :   Randomly chooses a new state up or down, then uses the Metropolis critera to decide whether to accept or reject:
            Min{1,exp(-(beta\_new u\_new - beta\_old u\_old)}

        **barker-transition**
        :   Randomly chooses a new state up or down, then uses the Barker transition critera to decide whether to accept or reject: exp(-beta\_new u\_new)/[exp(-beta\_new u\_new)+exp(-beta\_old u\_old)]

        **gibbs**
        :   Uses the conditional weights of the state given the coordinate (exp(-beta\_i u\_i) / sum\_k exp(beta\_i u\_i) to
            decide which state to move to.

        **metropolized-gibbs**
        :   :   Uses the conditional weights of the state given the coordinate (exp(-beta\_i u\_i) / sum\_k exp(beta\_i u\_i) to
                decide which state to move to, EXCLUDING the current state, then uses a rejection step to ensure detailed
                balance. Always more efficient that Gibbs, though only marginally so in many situations, such as when only the nearest neighbors have decent phase space overlap.

        **lmc-seed:**
        :   random seed to use for Monte Carlo moves in state space. If not specified, **ld-seed** is used instead.

        **mc-temperature:**
        :   Temperature used for acceptance/rejection for Monte Carlo moves. If not specified, the temperature of the
            simulation specified in the first group of **ref\_t** is used.

        **wl-ratio: (0.8)**
        :   The cutoff for the histogram of state occupancies to be reset, and the free energy incrementor to be reset as delta -> delta\*wl-scale. If we define the Nratio = (number of samples at each histogram) / (average number of samples at each histogram). **wl-ratio** of 0.8 means that means that the histogram is only considered flat if all Nratio > 0.8 AND simultaneously all 1/Nratio > 0.8.

        **wl-scale: (0.8)**
        :   Each time the histogram is considered flat, then the current value of the Wang-Landau incrementor for the free energies is multiplied by **wl-scale**. Value must be between 0 and 1.

        **init-wl-delta: (1.0)**
        :   The initial value of the Wang-Landau incrementor in kT. Some value near 1 kT is usually most efficient, though sometimes a value of 2-3 in units of kT works better if the free energy differences are large.

        **wl-oneovert: (no)**
        :   Set Wang-Landau incrementor to scale with 1/(simulation time) in the large sample limit. There is significant evidence that the standard Wang-Landau algorithms in state space presented here result in free energies getting 'burned in' to incorrect values that depend on the initial state. when **wl-oneovert** is true, then when the incrementor becomes less than 1/N, where N is the mumber of samples collected (and thus proportional to the data collection time, hence '1 over t'), then the Wang-Lambda incrementor is set to 1/N, decreasing every step. Once this occurs, **wl-ratio** is ignored, but the weights will still stop updating when the equilibration criteria set in **lmc-weights-equil** is achieved.

        **lmc-repeats: (1)**
        :   Controls the number of times that each Monte Carlo swap type is performed each iteration. In the limit of large numbers of Monte Carlo repeats, then all methods converge to Gibbs sampling. The value will generally not need to be different from 1.

        **lmc-gibbsdelta: (-1)**
        :   Limit Gibbs sampling to selected numbers of neighboring states. For Gibbs sampling, it is sometimes inefficient to perform Gibbs sampling over all of the states that are defined. A positive value of **lmc-gibbsdelta** means that only states plus or minus **lmc-gibbsdelta** are considered in exchanges up and down. A value of -1 means that all states are considered. For less than 100 states, it is probably not that expensive to include all states.

        **lmc-forced-nstart: (0)**
        :   Force initial state space sampling to generate weights. In order to come up with reasonable initial weights, this setting allows the simulation to drive from the initial to the final lambda state, with **lmc-forced-nstart** steps at each state before moving on to the next lambda state. If **lmc-forced-nstart** is sufficiently long (thousands of steps, perhaps), then the weights will be close to correct. However, in most cases, it is probably better to simply run the standard weight equilibration algorithms.

            **nst-transition-matrix: (-1)**
            :   Frequency of outputting the expanded ensemble transition matrix. A negative number means it will only be printed at the end of the simulation.:   **symmetrized-transition-matrix: (no)**
                    :   Whether to symmetrize the empirical transition matrix. In the infinite limit the matrix will be symmetric, but will diverge with statistical noise for short timescales. Forced symmetrization, by using the matrix T\_sym = 1/2 (T + transpose(T)), removes problems like the existence of (small magnitude) negative eigenvalues.

                    **mininum-var-min: (100)**
                    :   The **min-variance** strategy (option of **lmc-stats** is only valid for larger number of samples, and can get stuck if too few samples are used at each state. **mininum-var-min** is the minimum number of samples that each state that are allowed before the **min-variance** strategy is activated if selected.

                    **init-lambda-weights:**
                    :   The initial weights (free energies) used for the expanded ensemble states. Default is a vector of zero weights. format is similar to the lambda vector settings in **fep-lambdas**, except the weights can be any floating point number. Units are kT. Its length must match the lambda vector lengths.

                    **lmc-weights-equil: (no)**
                    :   **no**
                        :   Expanded ensemble weights continue to be updated throughout the simulation.

                        **yes**
                        :   The input expanded ensemble weights are treated as equilibrated, and are not updated throughout the simulation.

                        **wl-delta**
                        :   Expanded ensemble weight updating is stopped when the Wang-Landau incrementor falls below the value specified by **weight-equil-wl-delta**.

                        **number-all-lambda**
                        :   Expanded ensemble weight updating is stopped when the number of samples at all of the lambda states is greater than the value specified by **weight-equil-number-all-lambda**.

                        **number-steps**
                        :   Expanded ensemble weight updating is stopped when the number of steps is greater than the level specified by **weight-equil-number-steps**.

                        **number-samples**
                        :   Expanded ensemble weight updating is stopped when the number of total samples across all lambda states is greater than the level specified by **weight-equil-number-samples**.

                        **count-ratio**
                        :   Expanded ensemble weight updating is stopped when the ratio of samples at the least sampled lambda state and most sampled lambda state greater than the value specified by **weight-equil-count-ratio**.

                        **simulated-tempering: (no)**
                        :   Turn simulated tempering on or off. Simulated tempering is implemented as expanded ensemble sampling with different temperatures instead of different Hamiltonians.

                        **sim-temp-low: (300)**
                        :   Low temperature for simulated tempering.

                        **sim-temp-high: (300)**
                        :   High temperature for simulated tempering.

                        **simulated-tempering-scaling: (linear)**
                        :   Controls the way that the temperatures at intermediate lambdas are calculated from the **temperature-lambda** part of the lambda vector.
                        :   **linear**
                            :   Linearly interpolates the temperatures using the values of **temperature-lambda**,i.e. if **sim-temp-low**=300, **sim-temp-high**=400, then lambda=0.5 correspond to a temperature of 350. A nonlinear set of temperatures can always be implemented with uneven spacing in lambda.

                            **geometric**
                            :   Interpolates temperatures geometrically between **sim-temp-low** and **sim-temp-high**. The i:th state has temperature **sim-temp-low** \* (**sim-temp-high**/**sim-temp-low**) raised to the power of (i/(ntemps-1)). This should give roughly equal exchange for constant heat capacity, though of course things simulations that involve protein folding have very high heat capacity peaks.

                            **exponential**
                            :   Interpolates temperatures exponentially between **sim-temp-low** and **sim-temp-high**. The ith state has temperature
                                **sim-temp-low** + (**sim-temp-high**-**sim-temp-low**)\*((exp(**temperature-lambdas**[i])-1)/(exp(1.0)-1)).

  


---

### Non-equilibrium MDnon-equilibrium MD

**acc-grps:**
:   groups for constant acceleration (e.g.: Protein Sol)
    all atoms in groups Protein and Sol will experience constant acceleration
    as specified in the **accelerate** line

**accelerate: (0) [nm ps^-2^]**
:   acceleration for **acc-grps**; x, y and z for each group
    (e.g. 0.1 0.0 0.0 -0.1 0.0 0.0 means that first group has constant
    acceleration of 0.1 nm ps^-2^ in X direction, second group the
    opposite).

**freezegrps:**
:   Groups that are to be frozen (i.e. their X, Y, and/or Z position will
    not be updated; e.g. Lipid SOL). **freezedim** specifies for
    which dimension the freezing applies.
    To avoid spurious contibrutions to the virial and pressure due to large
    forces between completely frozen atoms you need to use
    energy group exclusions, this also saves computing time.
    Note that coordinates of frozen atoms are not scaled by pressure-coupling
    algorithms.

**freezedim:**
:   dimensions for which groups in **freezegrps** should be frozen,
    specify Y or N for X, Y and Z and for each group
    (e.g. Y Y N N N N means that particles in the first group
    can move only in Z direction. The particles in the second group can
    move in any direction).

**cos-acceleration: (0) [nm ps^-2^]**
:   the amplitude of the acceleration profile for calculating the
    viscosity.
    The acceleration is in the X-direction and the magnitude is
    **cos-acceleration** cos(2 pi z/boxheight).
    Two terms are added to the energy file:
    the amplitude of the velocity profile and 1/viscosity.

**deform: (0 0 0 0 0 0) [nm ps^-1^]**
:   The velocities of deformation for the box elements:
    a(x) b(y) c(z) b(x) c(x) c(y). Each step the box elements
    for which **deform** is non-zero are calculated as:
    box(ts)+(t-ts)\*deform, off-diagonal elements are corrected
    for periodicity. The coordinates are transformed accordingly.
    Frozen degrees of freedom are (purposely) also transformed.
    The time ts is set to t at the first step and at steps at which
    x and v are written to trajectory to ensure exact restarts.
    Deformation can be used together with semiisotropic or anisotropic
    pressure coupling when the appropriate compressibilities are set to zero.
    The diagonal elements can be used to strain a solid.
    The off-diagonal elements can be used to shear a solid
    or a liquid.

  


---

### Electric fieldselectric field

**E-x ; E-y ; E-z:**
:   If you want to use an electric field in a direction, enter 3 numbers
    after the appropriate **E-\***, the first number: the number of cosines,
    only 1 is implemented (with frequency 0) so enter 1,
    the second number: the strength of the electric field in
    **V nm^-1^**,
    the third number: the phase of the cosine, you can enter any number here
    since a cosine of frequency zero has no phase.

**E-xt; E-yt; E-zt:**
:   not implemented yet

  


---

  

### Mixed quantum/classical molecular dynamics

**QMMM:**
:   **no**
    :   No QM/MM.

    **yes**
    :   Do a QM/MM simulation. Several groups can be described at
        different QM levels separately. These are specified in
        the **QMMM-grps** field separated by spaces. The level of *ab
        initio* theory at which the groups are described is specified
        by **QMmethod** and **QMbasis** Fields. Describing the
        groups at different levels of theory is only possible with the ONIOM
        QM/MM scheme, specified by **QMMMscheme**.

**QMMM-grps:**
:   groups to be descibed at the QM level

**QMMMscheme:**
:   **normal**
    :   normal QM/MM. There can only be one **QMMM-grps** that is modelled
        at the **QMmethod** and **QMbasis** level of *ab initio*
        theory. The rest of the system is described at the MM level. The QM
        and MM subsystems interact as follows: MM point charges are included
        in the QM one-electron hamiltonian and all Lennard-Jones interactions
        are described at the MM level.

    **ONIOM**
    :   The interaction between the subsystem is described using the ONIOM
        method by Morokuma and co-workers. There can be more than one **QMMM-grps** each modeled at a different level of QM theory
        (**QMmethod** and **QMbasis**).

**QMmethod: (RHF)**
:   Method used to compute the energy and gradients on the QM
    atoms. Available methods are AM1, PM3, RHF, UHF, DFT, B3LYP, MP2,
    CASSCF, and MMVB. For CASSCF, the number of electrons and orbitals
    included in the active space is specified by **CASelectrons**
    and **CASorbitals**.

**QMbasis: (STO-3G)**
:   Basis set used to expand the electronic wavefuntion. Only Gaussian
    basis sets are currently available, *i.e.* STO-3G, 3-21G, 3-21G\*,
    3-21+G\*, 6-21G, 6-31G, 6-31G\*, 6-31+G\*, and 6-311G.

**QMcharge: (0) [integer]**
:   The total charge in e of the **QMMM-grps**. In case
    there are more than one **QMMM-grps**, the total charge of each
    ONIOM layer needs to be specified separately.

**QMmult: (1) [integer]**
:   The multiplicity of the **QMMM-grps**. In case there are more
    than one **QMMM-grps**, the multiplicity of each ONIOM layer needs
    to be specified separately.

**CASorbitals: (0) [integer]**
:   The number of orbitals to be included in the active space when
    doing a CASSCF computation.

**CASelectrons: (0) [integer]**
:   The number of electrons to be included in the active space when
    doing a CASSCF computation.

**SH:**
:   **no**
    :   No surface hopping. The system is always in the electronic
        ground-state.

    **yes**
    :   Do a QM/MM MD simulation on the excited state-potential energy
        surface and enforce a *diabatic* hop to the ground-state when the
        system hits the conical intersection hyperline in the course the
        simulation. This option only works in combination with the CASSCF
        method.

  


---

### Implicit solvent

**implicit-solvent:**
:   **no**
    :   No implicit solvent

    **GBSA**
    :   Do a simulation with implicit solvent using the Generalized Born formalism.
        Three different methods for calculating the Born radii are available, Still, HCT and
        OBC. These are specified with the **gb-algorithm** field. The non-polar solvation
        is specified with the **sa-algorithm** field.

    **gb-algorithm:**
    :   **Still**
        :   Use the Still method to calculate the Born radii

        **HCT**
        :   Use the Hawkins-Cramer-Truhlar method to calculate the Born radii

        **OBC**
        :   Use the Onufriev-Bashford-Case method to calculate the Born radii

        **nstgbradii: (1) [steps]**
        :   Frequency to (re)-calculate the Born radii. For most practial purposes,
            setting a value larger than 1 violates energy conservation and leads to
            unstable trajectories.

        **rgbradii: (1.0) [nm]**
        :   Cut-off for the calculation of the Born radii. Currently must be equal to rlist

        **gb-epsilon-solvent: (80)**
        :   Dielectric constant for the implicit solvent

        **gb-saltconc: (0) [M]**
        :   Salt concentration for implicit solvent models, currently not used

        **gb-obc-alpha (1); gb-obc-beta (0.8); gb-obc-gamma (4.85);**
        :   Scale factors for the OBC model. Default values are OBC(II).
            Values for OBC(I) are 0.8, 0 and 2.91 respectively

        **gb-dielectric-offset: (0.009) [nm]**
        :   Distance for the di-electric offset when calculating the Born radii. This is
            the offset between the center of each atom the center of the polarization energy
            for the corresponding atom

        **sa-algorithm**
        :   **Ace-approximation**
            :   Use an Ace-type approximation (default)

            **None**
            :   No non-polar solvation calculation done. For GBSA only the polar part gets
                calculated

            **sa-surface-tension: [kJ mol^-1^ nm^-2^]**
            :   Default value for surface tension with SA algorithms. The default value is -1;
                Note that if this default value is not changed
                it will be overridden by grompp using values that are specific for the choice
                of radii algorithm (0.0049 kcal/mol/Angstrom^2^ for Still, 0.0054 kcal/mol/Angstrom^2^
                for HCT/OBC)
                Setting it to 0 will while using an sa-algorithm other than None means
                no non-polar calculations are done.

  


---

### Adaptive Resolution Simulation

**adress: (no)**
:   Decide whether the AdResS feature is turned on.

**adress-type: (Off)**
:   **Off**
    :   Do an AdResS simulation with weight equal 1, which is equivalent to an explicit (normal) MD simulation. The difference to disabled AdResS is that the AdResS variables are still read-in and hence are defined.

    **Constant**
    :   Do an AdResS simulation with a constant weight, **adress-const-wf** defines the value of the weight

    **XSplit**
    :   Do an AdResS simulation with simulation box split in x-direction, so basically the weight is only a function of the x coordinate and all distances are measured using the x coordinate only.

    **Sphere**
    :   Do an AdResS simulation with spherical explicit zone.

**adress-const-wf: (1)**
:   Provides the weight for a constant weight simulation (**adress-type**=Constant)

**adress-ex-width: (0)**
:   Width of the explicit zone, measured from **adress-reference-coords**.

**adress-hy-width: (0)**
:   Width of the hybrid zone.

**adress-reference-coords: (0,0,0)**
:   Position of the center of the explicit zone. Periodic boundary conditions apply for measuring the distance from it.

**adress-cg-grp-names**
:   The names of the coarse-grained energy groups. All other energy groups are considered explicit and their interactions will be automatically excluded with the coarse-grained groups.

**adress-site: (COM)**The mapping point from which the weight is calculated.
:   **COM**
    :   The weight is calculated from the center of mass of each charge group.

    **COG**
    :   The weight is calculated from the center of geometry of each charge group.

    **Atom**
    :   The weight is calculated from the position of 1st atom of each charge group.

    **AtomPerAtom**
    :   The weight is calculated from the position of each individual atom.

**adress-interface-correction: (Off)**
:   **Off**
    :   Do not a apply any interface correction.

    **thermoforce**
    :   Apply thermodynamic force interface correction. The table can be specified using the -tabletf option of mdrun. The table should contain the potential and force (acting on molecules) as function of the distance from **adress-reference-coords**.

**adress-tf-grp-names**
:   The names of the energy groups to which the **thermoforce** is applied if enabled in **adress-interface-correction**. If no group is given the default table is applied.

**adress-ex-forcecap: (0)**
:   Cap the force in the hybrid region, useful for big molecules. 0 disables force capping.

  


---

### User defined thingies

**user1-grps; user2-grps:**

**userint1 (0); userint2 (0); userint3 (0); userint4 (0)**

**userreal1 (0); userreal2 (0); userreal3 (0); userreal4 (0)**
:   These you can use if you modify code. You can pass integers and
    reals to your subroutine. Check the inputrec definition in
    src/include/types/inputrec.h

  


---

### Index

acc-grps  
accelerate  
annealing  
annealing-npoints  
annealing-time  
annealing-temp  
bd-fric  
bDispCorr  
comm-mode  
comm-grps  
compressibility  
constraint-algorithm  
constraints  
cos-acceleration  
coulombtype  
coulomb-modifier  
couple-intramol  
couple-lambda0  
couple-lambda1  
couple-moltype  
cutoff-scheme  
define  
deform  
delta-lambda  
disre  
disre-weighting  
disre-mixed  
disre-fc  
disre-tau  
dt  
emstep  
emtol  
energygrp-excl  
energygrp-table  
energygrps  
epsilon-r  
epsilon-rf  
ewald-rtol  
ewald-geometry  
epsilon-surface  
E-x  
E-xt  
E-y  
E-yt  
E-z  
E-zt   
fcstep  
fourier-nx  
fourier-ny  
fourier-nz  
fourierspacing  
free-energy  
freezedim   
freezegrps  
gen-seed  
gen-temp  
gen-vel  
include  
init-lambda  
init-lambda-weights  
init-step  
initial-wl-delta  
integrator  
ld-seed  
lincs-iter  
lincs-order  
lincs-warnangle  
lmc-forced-nstart  
lmc-gibbsdelta  
lmc-mc-move  
lmc-seed  
lmc-stats  
lmc-weights-equil  
mc-temperature  
mininum-var-min  
morse  
nbfgscorr  
niter  
nh-chain-length  
nstcgsteep  
nstcalcenergy  
nstcomm  
nstdisreout  
nstenergy  
nsteps  
nstfout  
nstlist  
nstlog  
nstpcouple  
nsttcouple  
nstvout  
nstxout  
nstxtcout  
nst-transition-matrix  
ns-type  
nwall  
optimize-fft  
orire  
orire-fc  
orire-tau  
orire-fitgrp  
nstorireout  
pbc  
pcoupl  
pcoupltype  
periodic-molecules  
pme-order  
pull  
refcoord-scaling  
ref-p  
ref-t  
rcoulomb-switch  
rcoulomb  
rlist  
rlistlong  
rtpi  
rvdw-switch  
rvdw  
sc-alpha  
sc-power  
sc-sigma  
shake-tol  
sim-temp-low  
sim-temp-high  
simulated-tempering  
simulated-tempering-scaling  
symmetrized-transition-matrix  
table-extension  
tau-p  
tau-t  
tc-grps  
tcoupl  
tinit  
continuation  
user1-grps  
user2-grps  
userint1  
userint2  
userint3  
userint4  
userreal1  
userreal2  
userreal3  
userreal4  
vdwtype  
vdw-modifier  
verlet-buffer-drift  
xtc-grps  
xtc-precision  
zero-temp-time  
wall-atomtype  
wall-density  
wall-ewald-zfac  
wall-r-linpot  
wall-type  
weight-equil-count-ratio  
weight-equil-number-all-lambda  
weight-equil-number-samples  
weight-equil-number-steps  
weight-equil-wl-delta  
wl-ratio  
wl-scale  


---

http://www.gromacs.org
